# Mental well-being in young people with psychiatric disorders during the early phase of COVID-19 lockdown

**DOI:** 10.1371/journal.pone.0270644

**Published:** 2022-06-30

**Authors:** Emilie Orfeuvre, Nicolas Franck, Julien Plasse, Frédéric Haesebaert

**Affiliations:** 1 Centre Ressource de Réhabilitation Psychosociale, CH Le Vinatier, Lyon, France; 2 CH Le Vinatier, Pôle Centre Rive Gauche, Bron, France; 3 UMR 5229, CNRS & Université Lyon 1, Université de Lyon, Lyon, France; 4 SUR-CL3R-PEPS, CH Vinatier, Bron, Rhône-Alpes, France; 5 INSERM U1028, CNRS, UMR5292, PSYR2 Team, Lyon Neuroscience Research Center, Université Claude Bernard Lyon 1, Lyon, France; Washington University, St. Louis, UNITED STATES

## Abstract

**Background:**

Mental health and well-being were seriously impacted by the COVID-19 lockdown especially among young people and people with psychiatric disorders. This study aimed to identify factors associated with well-being in young people with psychiatric disorders, during early phase of COVID-19 lockdown in France.

**Methods:**

A national cross-sectional online study started on the 8^th^ day of COVID-19 lockdown in France (during March 25–30, 2020). We included young people aged from 16 to 29 who responded to the questionnaire, living and being confined in France, with past or current psychiatric treatment. The questionnaire was accessible online and explored demographics and clinical factors, well-being, stress, situation during lockdown. Well-being was measured by the Warwick-Edinburg Mental Well-Being Scale (WEMWBS). Simple and multiple linear regression analyses were carried out.

**Results:**

439 individuals were included with 262 (59.7%) previously treated and 177 (40.3%) currently treated. WEMWBS total score were 42.48 (9.05). Feeling of useful was the most affected dimension. Well-being was positively correlated with: currently working on site, physical activity, abilities to cope with difficulties, family and social supports (p<0.05). It was negatively correlated with: elevated stress level, anxious ruminations, dissatisfaction with information, difficulties to sleep or reorganize daily life, feeling supported by medicines (p<0.05). No individual factor was correlated with well-being. The stepwise linear multivariate model had simple R^2^ coefficient of determination of 0.535.

**Conclusion:**

In the specific population of young people with psychiatric disorders, factors associated with well-being at early stage of lockdown were mainly psychosocial and related to brutal disorganisation of daily life.

## Introduction

Well-being is a complex concept that combines eudaimonic and hedonic components. Eudaimonic or psychological well-being includes six main dimensions: self-acceptance, personal growth, purpose in life, positive relations with others, environmental mastery, autonomy [1]. Hedonic or subjective well-being refers to satisfaction with life and positive emotions [2]. Both perspectives refer to positive psychology that, by focusing on satisfactory aspects of daily life, psychological skills and needs, increases the ability to act and adapt to different events.

In December 2019, first cases of Coronavirus Disease 2019 (COVID-19) were diagnosed in Wuhan, China. On 11 March 2020, the World Health Organization (WHO) characterized the COVID-19 as a pandemic. To prevent the rapid spread of the virus, stringent nationwide lockdown was decided in France on March 16, 2020. The stress was sudden, major and multifactorial: physical distancing, loneliness, disorganization of daily life with inactivity and boredom, financial losses added to fears of infection, uncertain future and ruminations related to inadequate information.

As in previous pandemics [3], mental health was strongly impacted [4] with increased prevalence of anxious, depressive and post-traumatic symptoms [5] and aggravation of pre-existing psychiatric disorders [6]. Psychiatric symptoms and distress were more frequent and severe in very vulnerable populations, including young people and people with pre-existing psychiatric disorders [5,7–9], due to their high stress vulnerability [10]. Several alerts have been issued since the beginning of the pandemic on need for studies of these clinical subgroups to quickly develop early intervention strategies in mental health [11,12].

Disruption and disorganisation of daily life, caused by this brutal confinement, gave impression of new reality, new life which might be compared to occurrence of disease associated with functional alteration. Recovery-oriented approaches, aiming to achieve well-being despite illness, could also be interesting for everyone during such traumatic or stressful events. On the basis of their own goals, strengths and abilities, progressively, person regain pleasant, meaningful and engaged life [13]. Efficient, early and person-centred intervention requires identification of modifiable and causally well-being factors that could be different among different vulnerable subgroups [11].

Our study aimed at identifying factors associated with well-being in young people with psychiatric disorders, during the early phase of COVID-19 lockdown in France.

## Materials and methods

### Design and procedure

The data set came from our cross-sectional national, online observational study “LockUwell” [14], initiated on March 25, 2020, which aimed at studying mental wellbeing during the lockdown in France. The protocol respected the CHERRIES checklist (*Checklist for Reporting Results of Internet E-Surveys*) [15].

## Materials and data collection

The questionnaire was accessible online via web link, distributed on social networks, online media and mailing lists. Participation was voluntary, without counterpart or sampling. The time to answer was estimated to be between 15 to 30 minutes and the questionnaire could be completed in several times. The platform used was that of INSERM (*National Institute on Health and Mental Research)*. Only one response was possible per Internet Protocol address, to limit multiple responses. It was constructed, with a first and a second version, and available in English in supplementary material. The initial version consisted of 63 questions, quantitative and quantitative, single or multiple choices, divided into 6 domains: (a) Sociodemographic factors, (b) Level of well-being, (c) Level of stress, (d) Medical history with particular emphasis in psychiatric, psychological and addictological histories, (e) Perceptions of the COVID-19 pandemic and lockdown, (f) Lockdown process. Well-being was assessed by the Warwick-Edinburg Mental Well-Being Scale (WEMWBS) [16], translated and validated in French, and with excellent internal consistency [17]. The instrument refers to the last two weeks and consists of 14 items, evaluated according to a 5-point scale, the sum of which leads to an overall score ranging from 14 to 70 with higher scores associated with higher well being (no threshold exists for a state of well being, a former study indicated a mean score of 51.88 in a French student population [17]). A 11-point scale was used for the stress. A cut-off point at 6 were considered for “*severe stress*”. Tables 1 and 2 show relevant questions selected by authorships.

**Table 1:** Demographic and clinical characteristics of the whole sample (N = 439) and WEMWBS total scores.

	Number (%) of respondents	WEMWBS total score (Mean ± SD)
Sex		
Male	87 (19.8)	43.32 *±* 10.41
Female	335 (76.3)	42.5 *±* 8.72
Other	17 (3.9)	37.65 *±* 6.56
Age (year)		
16–17	16 (3.6)	39.31 *±* 11.94
18–19	32 (7.3)	41.06 *±* 10.62
20–24	144 (32.8)	41.11 *±* 8.59
25–29	247 (56.3)	43.66 *±* 8.74
Marital status		
Single, divorced, separated or widowed	220 (50.1)	41.42 *±* 9.29
With a partner	219 (49.9)	43.54 *±* 8.68
Parental status		
No child	421 (95.9)	42.52 *±* 9.03
One or more children	17 (3.9)	41 *±* 9.85
Work situation		
Other	219 (49.9)	40.41 *±* 9.26
Employed or independant worker	220 (50.1)	44.54 *±* 8.35
Student status		
Not student	242 (55.1)	43.1 *±* 9.06
Student	197 (44.9)	41.71 *±* 8.99
Education level (ISCED 2011)		
4 or less	113 (25.7)	39.75 *±* 10.11
5	52 (11.8)	42.46 *±* 7.38
6	91 (20.7)	41.71 *±* 8.77
7	143 (32.6)	44.55 *±* 8.43
8	40 (9.1)	44.55 *±* 8.66
Chronic illness or disability		
No	292 (66.5)	43.28 *±* 8.6
Yes	147 (33.5)	40.89 *±* 9.7
Psychiatric treatment		
Current	177(40.3)	40.64 *±* 9.31
Past	262 (59.7)	43.72 *±* 8.66
Ongoing addiction or psychological treatment		
No	288 (65.6)	43.15 *±* 8.99
Yes	151 (34.4)	41.19 *±* 9.04
Anxio-depressive disorders		
No	32 (7.3)	45.84 *±* 9.62
Yes	407 (92.7)	42.21 *±* 8.96
Sleep disorders		
No	286 (65.1)	43.32 *±* 9.25
Yes	153 (34.9)	40.91 *±* 8.47
Addiction		
No	395 (90.0)	42.46 *±* 9.18
Yes	44 (10.0)	42.64 *±* 7.78
Psychotic disorders		
Non	421 (95.9)	42.47 *±* 8.96
Yes	18 (4.1)	42.61 *±* 11.23
Eating disorders		
No	327 (74.5)	42.89 *±* 9.35
Yes	112 (25.5)	41.28 *±* 7.99
Neurodevelopmental disorders		
No	372 (84.7)	42.72 *±* 8.96
Yes	67 (15.3)	41.15 *±* 9.49

Abbreviations: WEMWBS, Warwick-Edinburg Mental Well-Being Scale; ISCED, International Standard Classification of Education.

**Table 2:** Situation during the COVID-19 lockdown and WEMWBS total scores.

	Number (%) of respondents	WEMWBS total score (Mean ± SD)
Overall stress level		
Weak (< 6)	148 (33.7)	47.28 ± 8.89
Elevated (> = 6)	291 (66.3)	40.04 ± 8.11
Agreement with lockdown measure		
Agree	400 (91.1)	42.72 ± 8.96
Neither agree nor disagree	20 (4.6)	38.65 ± 12.72
Disagree	19 (4.3)	37.32 ± 8.45
Satisfaction with the level of information		
Satisfied	230 (52.4)	44.61 ± 8.64
Neither satisfied nor dissatisfied	81 (18.5)	41.27 ± 9.21
Not satisfied	128 (9.2)	39.41 ± 8.69
Contact with any person(s) likely to be contaminated		
Being contaminated	32 (7.3)	45.03 ± 9.17
Being in direct contact with contaminated or likely to be contaminated person(s)	52 (11.8)	42.71 ± 8.9
Being not in direct contact with contaminated or likely to be contaminated person(s)	355 (80.9)	42.21 ± 9.04
Lockdown in usual accommodation		
Yes	355 (80.9)	42.43 (8.87)
No	84 (19.1)	42.67 (9.82)
Dwelling surface area (in m^2^)		
< = 29 m^2^	44 (10.0)	41.57 ± 9.01
30–59 m^2^	133 (30.3)	42.81 ± 8.57
60–89 m^2^	115 (26.2)	42.94 ± 8.83
> = 90 m^2^	141 (32.1)	42.23 ± 9.58
Outdoor space		
No	232 (52.8)	42.62 ± 8.85
Yes	207 (47.2)	42.32 ± 9.27
Number of people lockdown in household		
1	91 (20.7)	42.73 ± 8.91
2	178 (40.5)	43.58 ± 8.8
3–10	166 (37.8)	41.34 ± 9.2
Having one or all of your children living with you		
No	422 (96.1)	42.54 ± 9.02
Yes	17 (3.9)	41 ± 9.85
Working during lockdown		
Working on site	70 (15.9)	44.97 ± 8.74
Teleworking exclusively	192 (43.7)	43.35 ± 8.63
No professional activity	177 (40.3)	40.54 ± 9.26
Workload		
Decrease	87 (19.8)	44.84 ± 8.45
No change	65 (14.8)	42.78 ± 9.33
Increase	57 (13.0)	43.05 ± 8.17
Variable and unpredictable	53 (12.1)	44.08 ± 8.76
Risk of precarious situation		
Very likely	52 (11.8)	38.63 ± 9.09
Probably	64 (14.6)	42.47 ± 7.61
Probably not	171 (39.0)	41.76 ± 8.83
Certainly not	152 (34.6)	44.61 ± 9.34
Work or study		
Never	83 (18.9)	38.47 ± 9.78
Less than 30 minutes	45 (10.3)	42.71 ± 8.38
From 30 minutes to 1 hour	31 (7.1)	39.74 ± 8.2
More than 1 hour	280 (63.8)	43.93 ± 8.62
Take care of yourself		
Never	9 (2.1)	37 ± 9.91
Less than 30 minutes	213 (48.5)	40.14 ± 9.03
From 30 minutes to 1 hour	145 (33.0)	44.75 ± 8.38
More than 1 hour	72 (16.4)	45.51 ± 8.24
Nap		
Never	196 (44.6)	42.6 ± 9.17
Less than 30 minutes	67 (15.3)	43.72 ± 8.53
From 30 minutes to 1 hour	54 (12.3)	44.56 ± 8.15
More than 1 hour	122 (27.8)	40.68 ± 9.27
Read		
Never	106 (24.1)	40.17 ± 10.28
Less than 30 minutes	85 (19.4)	43.29 ± 8.16
From 30 minutes to 1 hour	109 (24.8)	42.6 ± 8.26
More than 1 hour	139 (31.7)	43.65 ± 8.91
Creative activities (music, drawing…)		
Never	164 (37.4)	41.91 ± 9.89
Less than 30 minutes	72 (16.4)	41.9 ± 8.53
From 30 minutes to 1 hour	80 (18.2)	42.94 ± 8.41
More than 1 hour	123 (28.0)	43.28 ± 8.58
Practice physical activities		
Never	168 (38.3)	39.68 ± 9.26
Less than 30 minutes	96 (21.9)	42.8 ± 8.68
From 30 minutes to 1 hour	103 (23.5)	44.61 ± 8.53
More than 1 hour	72 (16.4)	45.51 ± 7.95
Play video games		
Never	211 (48.1)	42.5 ± 9.09
Less than 30 minutes	33 (7.5)	41.48 ± 8.23
From 30 minutes to 1 hour	32 (7.3)	45.44 ± 8.31
More than 1 hour	163 (37.1)	42.07 ± 9.25
Ruminating or being the object of anxious fears		
Never	55 (12.5)	51.69 ± 7.94
Less than 30 minutes	104 (23.7)	45.76 ± 7.31
From 30 minutes to 1 hour	86 (19.6)	43.22 ± 6.93
More than 1 hour	194 (44.2)	37.78 ± 8.12
Difficulties in having good and regular sleep		
No	110 (25.1)	47.33 ± 8.77
Yes	329 (74.9)	40.86 ± 8.56
Difficulties in having regular alimentation		
No	152 (34.6)	44.74 ± 8.81
Yes	287 (65.4)	41.28 ± 8.95
Difficulties in establishing new routines		
No	232 (52.8)	44.36 ± 9.16
Yes	207 (47.2)	40.37 ± 8.46
Being helped by media		
No	301 (68.6)	41.66 ± 9.29
Yes	138 (31.4)	44.27 ± 8.24
Being helped by abilities to cope with difficulties		
No	144 (32.8)	39.33 ± 9.34
Yes	295 (67.2)	44.02 ± 8.5
Being helped by conviction of favourable outcome		
No	161 (36.7)	40.37 ± 10.02
Yes	278 (63.3)	43.7 ± 8.21
Being helped by religious faith		
No	403 (91.8)	42.34 ± 9.13
Yes	36 (8.2)	44 ± 8.06
Being helped by support		
No	198 (45.1)	41.33 ± 9.75
Yes	241 (54.9)	43.42 ± 8.33
Being helped by substances		
No	365 (83.1)	42.81 ± 9.22
Yes	74 (16.9)	40.86 ± 7.98
Being helped by medicines		
No	355 (80.9)	43.88 ± 8.81
Yes	84 (19.1)	36.55 ± 7.55
Coffee, tea, energy drinks use		
No use	73 (16.6)	40.23 ± 9.75
No change	203 (46.2)	43.77 ± 8.43
Decrease or cessation	33 (7.5)	43.24 ± 7.6
Increase	130 (29.6)	41.53 ± 9.63
Caloric food		
No use	12 (2.7)	45.5 ± 10.72
No change	165 (37.6)	43.96 ± 8.85
Decrease or cessation	52 (11.8)	41.06 ± 9.45
Increase	210 (47.8)	41.5 ± 8.86
Tobacco use		
No use	288 (65.6)	42.82 ± 9.36
No change	38 (8.7)	42.68 ± 8.52
Decrease or cessation	41 (9.3)	43.73 ± 5.47
Increase	72 (16.4)	40.28 ± 9.33
Alcohol use		
No use	181 (41.2)	40.96 ± 9.56
No change	99 (22.6)	43.79 ± 8.84
Decrease or cessation	77 (17.5)	43.58 ± 8.78
Increase	82 (18.7)	43.22 ± 7.97
Cannabis use		
No use	377 (85.9)	42.84 ± 9.15
No change	22 (5.0)	43.14 ± 8.55
Decrease or cessation	13 (3.0)	37 ± 9.83
Increase	27 (6.2)	39.52 ± 6.16
Other drugs (ecstasy, heroin, …)		
No use	418 (95.2)	42.55 ± 9.11
No change	8 (1.8)	42.88 ± 9.23
Decrease or cessation	10 (2.3)	40.8 ± 7.38
Increase	3 (0.7)	37.67 ± 5.51
Medicines use		
No use	195 (44.4)	44.21 ± 8.91
No change	123 (28.0)	41.93 ± 8.17
Decrease or cessation	24 (5.5)	46.17 ± 8.63
Increase	97 (22.1)	38.78 ± 9.3
Screens use		
No use	3 (0.7)	38.33 ± 9.24
No change	84 (19.1)	43.57 ± 8.91
Decrease or cessation	10 (2.3)	42.9 ± 11.26
Increase	342 (77.9)	42.23 ± 9.02
Face to face interactions		
Maximum once a week	370 (84.3)	42.34 ± 8.92
Several times a week	28 (6.4)	42.79 ± 10.36
Every day	41 (9.3)	43.49 ± 9.38
Phone or texting interactions		
Maximum once a week	29 (6.6)	38.17 ± 10.21
Several times a week	203 (46.2)	41.64 ± 8.5
Every day	207 (47.2)	43.9 ± 9.15
Social networks interactions		
Maximum once a week	76 (17.3)	39.17 ± 9.0
Several times a week	112 (25.5)	43.22 ± 9.24
Every day	251 (57.2)	43.15 ± 8.78
Support		
No	61 (13.9)	37.59 ± 9.94
Yes	378 (86.1)	43.27 ± 8.65
Family support		
No	93 (21.2)	37.94 ± 9.81
Yes	346 (78.8)	43.7 ± 8.44
Friend support		
No	140 (31.9)	39.29 ± 9.88
Yes	299 (68.1)	43.97 ± 8.23
Health or another professionals support		
No	352 (80.2)	42.78 ± 8.92
Yes	87 (19.8)	41.24 ± 9.49
Other social support (*colleagues*, *neighbours*, *associations*, *…)*		
No	286 (65.1)	40.42 ± 8.98
Yes	153 (34.9)	46.33 ± 7.85
Having pet		
No	213 (48.5)	41.97 ± 9.19
Yes	226 (51.5)	42.96 ± 8.9

Abbreviations: WEMWBS, Warwick-Edinburg Mental Well-Being Scale; COVID-19, Coronavirus Disease 2019.

### Participants

The inclusion criteria were: (1) age between 16 to 29 years old, (2) living and being confined in France, (3) a past or current psychiatric treatment. Only data from the 8^th^ to 13^th^ day of lockdown, i.e. from March 25, 2020 to March 30, 2020 were analysed, to could be compared with the previous analyses [14] and to limit possible time biases. During this period, participants for our study were selected among these above-mentioned population.

### Statistical analysis

The software R were used. Incomplete questionnaires were removed. No weighting of the data was performed due to the lack of reference to this specific population. Univariate and bivariate tests by analysis of variance (ANOVA) were performed. Multiple linear regression analyses were performed including candidate variable with a significant bivariate test with a p-value less than 0.1. Given the exploratory nature of the study, a stepwise method was preferred to a hierarchical or non-hierarchical “*forced entry*” method. The results were considered statistically significant if the p-value was less than 0.05.

### Ethics statement

The research board of the Vinatier Hospital (Bron, France) stated that no ethics committee approval was needed and that the project was conducted in accordance with survey ethics. Indeed, as the survey was conducted anonymously with no personal data the EU General Data Protection Regulation (GDPR) of May 25, 2018 did not apply.

## Results

### Sample characteristics

We analysed data from 439 eligible young people whose questionnaire was fully completed. Main sociodemographic and clinical characteristics were (see Table 1 for more details): 335 participants (76.3%) were female, their mean age was 24.53 (3.42) years and 247 of them (56.3%) were aged between 25 to 29 years. 219 (49.9%) were in couple and 17 (3.9%) had children. Main academic and professional characteristics were: 274 participants (62.4%) had university degree or higher (ISCED > = 6), 220 of them (50.1%) worked and 197 (44.9%) were student, which could be combined. As required by the inclusion criteria, all of them had benefited from psychiatric treatment and 177 (40.3%) were still treated; 151 (34.4%) had psychological or addiction treatment; 407 (92.7%) suffered from anxio-depressive disorders, 153 (34.9%) from sleep disorders and 18 (4.1%) from psychotic disorders. Many disorders might be associated.

### Lockdown processing

Main information related to lockdown were (see Table 2 for more details): 400 participants (91.1%) agreed with measures taken but 128 (29.2%) were unsatisfied with the information provided. 32 (7.3%) were infected; 207 (47.2%) had access to outdoor space and mean housing surface area was 79.73m2 (55.6); 91 (20.7%) were confined alone; 177 (40.3%) did not work and 70 (15.9%) left their house to go to work; 264 (60.1%) practiced less than 30 minutes of sport per day. Respectively 329 (74.9%), 287 (65.4%) and 207 (47.2%) individuals had difficulties sleeping, eating regularly and reorganizing their daily life. Abilities to cope with difficulties, positive consequences and support helped respectively 295 (67.2%), 278 (63.3%) and 241 (54.9%) of individuals to cope with the lockdown. Screen use and caloric food intake increased among 342 (77.9%) and 97 (22.1%) individuals respectively. 195 (44.4%) did not take medication and 97 (22.1%) of the individuals concerned increased their medication consumption. 84 (19.1%) of all participants felt helped by medications. Social networks and phones represented the two main vectors of social interactions, and were used daily by 251 (57.2%) and 207 (47.2%) individuals respectively. 378 (86.1%) received support, which was mainly family for 346 (78.8%), friendly for 299 (68.1%) and social for 153 (34.9%).

### Well-being and stress

Total mean WEMWBS score was 43.72 (±8.66) for the 262 (59.7%) individuals previously treated and 40.64 (±9.31) for the 177 (40.3%) still treated. Mean scores per variable were detailed in Tables 1 and 2. Feeling of useful was the dimension the most affected with an average of 2.32 (±1.05) as presented in Table 3. 291 (66.3%) of individuals were considered highly stressed (high score > = 6). 194 (44.2%) experienced anxious ruminations for more than one hour per day while 55 (12.5%) were not concerned.

**Table 3:** WEMWBS subscores during the COVID-19 lockdown.

Variables	Statements	No. (%) of respondents	Mean ± SD
1	To have been feeling optimistic		2.85 ± 0.96
	None of the time	34 (7.7)	
	Rarely	123 (28.0)	
	Some of the time	172 (39.21)	
	Often	94 (21.4)	
	All of the time	16 (3.6)	
2	To have been feeling useful		2.32 ± 1.05
	None of the time	109 (24.8)	
	Rarely	155 (35.3)	
	Some of the time	108 (24.6)	
	Often	60 (13.7)	
	All of the time	7 (1.6)	
3	To have been relaxed		2.86 ± 0.92
	None of the time	28 (6.4)	
	Rarely	123 (28.0)	
	Some of the time	183 (41.7)	
	Often	92 (21.0)	
	All of the time	13 (3.0)	
4	To have been interested in other people		3.51 ± 1.06
	None of the time	22 (5.0)	
	Rarely	56 (12.8)	
	Some of the time	109 (24.8)	
	Often	182 (41.5)	
	All of the time	70 (16.0)	
5	To have had energy to spare		3.22 ± 1.13
	None of the time	32 (7.3)	
	Rarely	86 (19.6)	
	Some of the time	135 (30.8)	
	Often	126 (28.7)	
	All of the time	60 (13.7)	
6	To have been dealing with problems well		3.12 ± 1.02
	None of the time	30 (6.8)	
	Rarely	89 (20.1)	
	Some of the time	144 (33.0)	
	Often	149 (29.6)	
	All of the time	27 (10.5)	
7	To have been thinking clearly		3.17 ± 1.08
	None of the time	30 (16.6)	
	Rarely	88 (29.2)	
	Some of the time	145 (31.4)	
	Often	130 (18.9)	
	All of the time	46 (10.5)	
8	To have been feeling good about yourself		2.64 ± 1.08
	None of the time	73 (16.6)	
	Rarely	128 (29.2)	
	Some of the time	138 (31.4)	
	Often	83 (18.9)	
	All of the time	17 (3.9)	
9	To have been feeling close to other people		3.02 ± 1.07
	None of the time	37 (8.4)	
	Rarely	110 (25.1)	
	Some of the time	126 (28.7)	
	Often	141 (32.1)	
	All of the time	25 (5.7)	
10	To have been feeling confident		2.62 ± 0.98
	None of the time	53 (12.1)	
	Rarely	150 (34.2)	
	Some of the time	156 (35.5)	
	Often	68 (15.5)	
	All of the time	12 (2.7)	
11	To have been able to make up your own mind about things		3.70 ± 0.94
	None of the time	8 (1.8)	
	Rarely	39 (8.9)	
	Some of the time	116 (26.4)	
	Often	190 (43.3)	
	All of the time	86 (19.6)	
12	To have been feeling loved		3.49 ± 1.05
	None of the time	18 (4.1)	
	Rarely	56 (12.8)	
	Some of the time	137 (31.2)	
	Often	150 (34.2)	
	All of the time	78 (17.8)	
13	To have been interested in new things		3.12 ± 1.10
	None of the time	35 (8.0)	
	Rarely	96 (21.9)	
	Some of the time	128 (29.2)	
	Often	140 (31.9)	
	All of the time	40 (9.1)	
14	To have been feeling cheerful		2.84 ± 0.92
	None of the time	35 (8.0)	
	Rarely	115 (26.2)	
	Some of the time	183 (41.7)	
	Often	99 (22.6)	
	All of the time	7 (1.6)	

Abbreviations: WEMWBS, Warwick-Edinburg Mental Well-Being Scale.

### Factors associated with well-being

The simple and multiple linear regression coefficients are presented in Tables 4 and 5. The factors positively correlated with well-being were: work at workplace, physical activity, abilites to cope with difficulties, family and social supports. Those negatively correlated were: elevated stress level, anxious ruminations, dissatisfaction with information provided, difficulties to sleep or reorganize daily life, feeling supported by medications. The physical activity was protector from 30 minutes per day and the effect increased with the duration of practice. Anxious ruminations were strongly and negatively correlated and the coefficients increased according their importance, estimated by daily durations. No individual factor was correlated with well-being. The stepwise linear multivariate model had a simple R^2^ coefficient of determination of 0.535.

**Table 4:** Factors associated with well-being during the COVID-19 lockdown along simple linear regression analysis.

Variables	N	eta2 (1)	p.value.F (2)	Parameters
Sex	439	0.013	1.000	Aov: F(2,436) = 2.828
Age	439	0.023	0.528	Aov: F(3,435) = 3.484
Marital status	439	0.014	0.476	Aov: F(1,437) = 6.076
Parental status	439	0.001	1.000	Aov: F(1,436) = 0.463
Work situation	439	0.052	0.000***	Aov: F(1,437) = 24.139
Student status	439	0.006	1.000	Aov: F(1,437) = 2.583
Education level	439	0.047	0.015*	Aov: F(4,434) = 5.322
Chronic illness or disability	439	0,016	0,315	Aov: F(1,437) = 6.896
Current psychiatric treatment	439	0,028	0,018*	Aov: F(1,437) = 12.595
Current psychological or addiction treatment	439	0,011	0,812	Aov: F(1,437) = 4.693
Anxio-depressive disorders	439	0,011	0,812	Aov: F(1,437) = 4.819
Sleep disorders	439	0,016	0,288	Aov: F(1,437) = 7.173
Addiction	439	0	1,000	Aov: F(1,437) = 0.015
Psychotic disorders	439	0	1,000	Aov: F(1,437) = 0.004
Eating disorders	439	0,006	1,000	Aov: F(1,437) = 2.663
Neurodevelopmental disorders	439	0,004	1,000	Aov: F(1,437) = 1.71
Overall stress level	439	0,143	0,000***	Aov: F(1,437) = 73.188
Agreement with the lockdown measure	439	0,024	0,190	Aov: F(2,436) = 5.461
Satisfaction with the level of information	439	0,066	0,000***	Aov : F(2,436) = 15.393
Contact with any person(s) likely to be contaminated	439	0,007	1,000	Aov : F(2,436) = 1.446
Lockdown in usual accommodation	439	0.001	0.832	Aov :F(1.437) = 0.045
Accommodation surface area, m^2^	433	0,002	1,000	Aov: F(3,429) = 0.342
Outdoor space	439	0,0003	1,000	Aov: F(1,437) = 0.122
Number of people lockdown in Household	435	0,012	1,000	Aov: F(2,432) = 2.685
Having a child lockdown with you	439	0,001	1,000	Aov: F(1,437) = 0.472
Work modalities	439	0,035	0,018*	Aov: F(2,436) = 7.85
Workload	262	0,01	1,000	Aov: F(3,258) = 0.87
Risk of precarious situation	439	0,043	0,011*	Aov: F(3,435) = 6.528
Work or study	439	0,06	0,000***	Aov: F(3,435) = 9.299
Take care of yourself	439	0,08	0,000***	Aov: F(3,435) = 12.534
Nap	439	0,02	0,812	Aov: F(3,435) = 3.028
Read	439	0,023	0,570	Aov: F(3,435) = 3.366
Creative activities (music, drawing, …)	439	0,005	1,000	Aov: F(3,435) = 0.7
Practice physical activities	439	0,068	0,000***	Aov: F(3,435) = 10.656
Play video games	439	0,009	1,000	Aov: F(3,435) = 1.39
Ruminating or being the object of anxious fears	439	0,282	0,000***	Aov: F(3,435) = 57.058
Difficulties in having good and regular sleep	439	0,096	0,000***	Aov: F(1,437) = 46.563
Difficulties in having regular alimentation	439	0,033	0,006**	Aov: F(1,437) = 15.045
Difficulties in establishing new routines	439	0,048	0,000***	Aov: F(1,437) = 22.27
Being helped by media	439	0,018	0,190	Aov: F(1,437) = 8.005
Being helped by abilities to cope with difficulties	439	0,059	0,000***	Aov: F(1,437) = 27.598
Being helped by conviction of favourable outcome	439	0,031	0,009**	Aov: F(1,437) = 14.192
Being helped by religious faith	439	0,003	1,000	Aov: F(1,437) = 1.11
Being helped by support	439	0,013	0,528	Aov: F(1,437) = 5.843
Being helped by substance	439	0,006	1,000	Aov: F(1,437) = 2.844
Being helped by medicines	439	0,102	0,000***	Aov: F(1,437) = 49.608
Coffee, tea and energetic drinks use	439	0,023	0,528	Aov: F(3,435) = 3.488
Caloric food	439	0,022	0,667	Aov: F(3,435) = 3.22
Tobacco use	439	0,013	1,000	Aov: F(3,435) = 1.839
Alcohol use	439	0,02	0,812	Aov: F(3,435) = 3.009
Cannabis use	439	0,019	0,912	Aov: F(3,435) = 2.829
Other drugs use (*ecstasy*, *heroin…)*	439	0,003	1,000	Aov: F(3,435) = 0.409
Medicines use	439	0,063	0,000***	Aov: F(3,435) = 9.798
Screens use	439	0,005	1,000	Aov: F(3,435) = 0.708
Face to face interactions	439	0,001	1,000	Aov: F(2,436) = 0.312
Phone or texting interactions	439	0,031	0,040*	Aov: F(2,436) = 6.907
Social networks interactions	439	0,028	0,078	Aov: F(2,436) = 6.295
Support	439	0,047	0,000***	Aov: F(1,437) = 21.664
Family support	439	0,068	0,000***	Aov: F(1,437) = 31.857
Friend support	439	0,058	0,000***	Aov: F(1,437) = 27.133
Health professionals support	439	0,005	1,000	Aov: F(1,437) = 2.034
Other social (*colleagues*, *neighbours*, *association*s…)	439	0,097	0,000***	Aov: F(1,437) = 47.146
Having Pet	439	0,003	1,000	Aov: F(1,437) = 1.322

*p-value<0.05

**p-value<0.01

***p-value<0.001.

(1) Effect size: 0.01–0.06 (low), 0.06–0.14 (medium) and > = 0.14 (high).

(2) Holm’s procedure.

Abbreviations: COVID-19, Coronavirus Disease of 2019.

**Table 5:** Factors associated with well-being during the COVID-19 lockdown along stepwise multiple linear regression analysis.

Variables	Estimates	CI 95%	Statistic	p
Intercept	48,04	43.13 – 52.96	19,2	<0.001***
Work Yes	1,07	-0.25 – 2.38	1,6	0,111
Education level Réf.: ISCED 4 or less				
ISCED 5	1,81	-0.28 – 3.91	1,7	0,089
ISCED 6	0,13	-1.65 – 1.92	0,15	0,885
ISCED 7	1,66	-0.03 – 3.35	1,93	0,054
ISCED 8	-0,7	-3.20 – 1.79	-0,55	0,581
Overall stress level Elevated > = 6	-3,06	-4.46 – -1.66	-4,3	<0.001***
Satisfaction with the level of information				
Neither satisfied nor dissatisfied	-1,55	-3.18 – 0.09	-1,86	0,064
Not satisfied	-1,95	-3.38 – -0.52	-2,68	0,008**
Working versus unworking	0,91	-0.00 – 1.81	1,97	0,05
Working on site versus telecommuting	0,9	0.01 – 1.80	1,98	0,049*
Take care of yourself				
Less than 30 minutes				
From 30 minutes to 1 hour	-1,64	-5.87 – 2.59	-0,76	0,447
More than 1 hour	1,46	-2.82 – 5.75	0,67	0,502
Practice physical activities	1,95	-2.49 – 6.39	0,86	0,388
Less than 30 minutes	0,98	-0.63 – 2.58	1,2	0,232
From 30 minutes to 1 hour	1,59	0.00 – 3.18	1,97	0,049*
More than 1 hour	3,05	1.21 – 4.88	3,26	0,001**
Ruminating or being the object of anxious fears				
Less than 30 minutes	-4,49	-6.59 – -2.40	-4,22	<0.001***
From 30 minutes to 1 hour	-5,98	-8.19 – -3.77	-5,32	<0.001***
More than 1 hour	-8,57	-10.73 – -6.42	-7,82	<0.001***
Difficulties in having good and regular sleep	-2,79	-4.21 – -1.37	-3,85	<0.001***
Difficulties in establishing new routines	-1,6	-2.81 – -0.38	-2,58	0,01*
Being helped by abilities to cope with difficulties	1,64	0.32 – 2.95	2,45	0,015*
Being helped by conviction of favourable outcome	1,17	-0.11 – 2.45	1,8	0,073
Being helped by medicines	-2,93	-4.53 – -1.33	-3,6	<0.001***
Family support	2,91	1.40 – 4.43	3,77	<0.001***
Other social support (colleagues, neighbours, associations…)	1,73	0.31 – 3.15	2,4	0,017*

Number of respondents 439.

Adjusted R^2^ / R^2^ 0.563 / 0.535.

AIC 2871, 164.

Stepwise AIC (vars cand p < 0.1).

*p-value<0.05

**p-value<0.01

***p-value<0.001.

Abbreviations: ISCED, International Standard Classification of Education, COVID-19, coronavirus disease 2019; AIC, Akaike Information Criterion.

## Discussion

This first online study aimed to identify factors associated with well-being, at the early stage of lockdown, in young people concerned by psychiatric disorders. It occurred within the context of psychological health emergency following the COVID-19 pandemic [11,12,18] and aimed at identifying targets for early intervention.

### Altered well-being in young people with psychiatric disorders

Studying well-being required caution because of lack of consensual definition of “*mental health”* and “*well-being*” [19] and multiplicity of psychometric tools. WEMWBS was chosen for its analysis of both hedonic and eudemonic aspects, over the last two weeks, with good internal consistency and reproducibility [17]. Although, to date, no baseline data on young people with psychiatric disorders were available, our results highlighted significative impairment of wellbeing, with unknown kinetics. Outside pandemic period, French study [17] reported WEMWBS mean score of 44.86 (9.22) among French people suffering from schizophrenia in recovery process. However, such a score, is only partially comparable due to heterogeneity of our sample and the low representation of psychotic disorders. Concerning young workers and students without psychiatric disorders, WEMWBS mean scores were higher of 51.47 (7.19) and 51.88 (6.87) respectively. Scottish study found median score for young people of 53 (IC 95% [52–53]) [16]. First global analysis of our dataset showed, by the second week of lockdown, lower well-being when compared to studies outside lockdown setting among young people, and people with past or actual psychiatric disorder with mean scores of 47.80 (7.23), 48.40 (8.52) and 45.02 (8.56) [14]. This early impairment of well-being was consistent with the onset of major distress and psychiatric symptoms during this period [4,20,21].

### Factors of well-being

Contrary to data in general population, no individual and pre-existing factors of well-being could be identified. All young people with psychiatric disorders, past or currently treated, and whatever the type of disorder, must be considered as at risk.

Severe stress and major anxiety were reported by 66.3% and 44.4% of young, in line with literature data showing higher levels in case of psychiatric history [8,20]. To date, major impact of stress in well-being were poorly documented during pandemic while its role on aggravation or onset of psychiatric disorders were established [5–7,22]. Being young or suffering from psychiatric disorders increased significantly risk of such psychiatric, but also physical, consequences [5,7,9], due to high vulnerability to stress [10,23].

At the early stage of brutal lockdown, many factors identified in our study refer to the suddenly break and disorganization of daily life. Bidirectional relationships between circadian rhythms and mental health were established in former studies [24]. The importance of routines and regular rhythms justifies psychoeducation of all young people to help them structure their daily life, creating new habits with regular sport and various activities, deciding on regular bedtime. Simple and individual timeframe might be helpful. Limiting late exposure to screens and permanent nibbling could facilitate falling asleep and restoration of dietary rhythms by reappearance of hunger and satiety signals.

Mental health benefits of regular physical activity were observed in general [25] and clinical [26] populations, and during COVID-19 pandemic [27]. Our study found the beneficial threshold of half an hour per day observed in Zhang’s study [27], who also noted negative correlation in case of excessive exercise for more than two and half hours per day, without specifying sense of cause-effect link.

Working did not impact well-being, before or during the lockdown but telecommuting was damaging. Interaction analysis could be interesting between working status, psychiatric status and well-being to understand such an indifference which could be the result of reduced interest in work related to recovery process or depressive symptomatology. In general population, stop working was associated with lower well-being at the early stage but not telecommuting. [14,27]. Surprisingly, in our study, studying did not increase the risk while it did in general population [5,14].

Our study highlighted that satisfaction with information promoted well-being. Overabundance of information, rumors and misinformation are classic in pandemic context [28] but should be controlled at the risk of serious physical, psychical and social consequences [29,30]. During pandemic periods, information quality strongly conditioned respect of health recommendations [31] and psychological consequences. Information should be clear, easy to access, and concordant between reference sources (government, other decision-making bodies, health professionals), especially for risk levels which easily generates fear [3,30]. At the individual level, the WHO recommended limiting time of exposure and favoring reliable and official sources [32]. Major and increasing use of screens in our study and in the general population made this limitation complex [33]. Information was everywhere, quickly spread and might be intrusive, appearing spontaneous through social networks, newsletters, internet sites… Active participation of young people was required to control rumors and media exposure, prevent panic and preserve well-being.

Variations in drug treatment did not interfere with well-being, but young who felt helped could need special attention. Although data on the safety were insufficient at this time [34], continuation of treatment was recommended because of excessive risk of aggravation of psychiatric disorders and withdrawal syndrome [35]. In our study, 97 (39.8%) young people increased their treatment, which could be related to an increase in psychiatric symptomatology [6].

### A person-centered approach

Promotion of well-being in pandemic period could be compared to recovery-oriented approach, requiring interdisciplinary collaboration and active participation of young people. Empowerment contributes to supporting eudemonic well-being by reinforcing senses of useful and control [36], self-esteem and self-confidence. After having evaluated risk, psychoeducational interventions could help young people to identify their vulnerabilities, harmful environmental factors, but also their coping skills, recovery strengths and environmental resources in order to boost resilience.

Psychoeducation must be proposed to entire family to promote adaptative family coping and cohesion in order to preserve family support. Communication must be warm, caring, regular and interesting [37]. Stress of lockdown added to burden of disease, leading to high-risk for mental health of caregivers who needed support themselves: impaired well-being, quality of life, depression, isolation and financial difficulties [37–39]. During lockdown, 50% of caregivers did not feel supported according to French survey by UNAFAM (*The French National Union of Families and Friends of Sick or Psychically Handicapped People)* [40].

Individual resilience and social support are highly related [41], which could contribute to protector effect of social support. Sense of cohesion should be strengthened by citizen involvement, neighbourhood solidarity, and promotion interactions, whatever their frequency and with respect for physical distancing measures. Respecting containment is already a responsible and altruistic act that should be valued. Continuing group therapeutic activities could be also interesting to maintaining peer relationships.

### Strengths and limitations

Firstly, our study could not analyse kinetics of degradation of well-being and variations over time of different studied variables because of its cross-sectional nature. A cohort study would have been ideal but none ethics committee could be mobilized very quickly in France in March 2020.

Secondly, several recruitment biases must be taken into account. Convenience sampling used for LockUwell survey could explain part of the over-representation of anxiety and depressive disorders and the under-representation of psychotic disorders. However, easy to carry out, it allowed us to quickly obtain a large sample. Need for access to digital technologies, existence of motivational factor due to absence of counterpart, choice of intermediate inclusion criteria also impacted representativeness. As inclusion depended on the presence of current or past psychiatric cares, young people who never engaged with services because of refusal, denial, lack of demand, difficulties in accessing care or non-reporting for fear of stigmatisation or coerced cares were not included. However, psychiatric cares were clinical and relevant criterion, focusing on severity rather than the type of mental disorder.

Thirdly, the setting of our survey, targeting general population with online response limited the level of precision in clinical explorations. Some specific variables to this population could be interesting to improve risk prediction at the start of lockdown: age of onset of disorder, addictions, medications, type of follow-up… Current absence of specific risk factors must encourage proactive contact, evaluation and closely support systematically for each young people suffering from psychiatric disorders.

## Conclusions

Several factors impacting well-being of young people with psychiatric disorders, at early stage of lockdown, have been identified. Mainly psychosocial and related to brutal disorganisation of daily life, these factors could justify early psychoeducational interventions aiming at boosting resilience, fostering empowerment and promoting social relationships.

## Supporting information

S1 File (XLS)

## Abbreviations

COVID-19: Coronavirus Disease of 2019
CI: Confidence Interval
ISCED: International Standard Classification of Education
SD: Standard Deviation
WEMWBS: Warwick-Edinburg Mental Wellbeing Scale

## References

[1] Ryff CD. (1989). Happiness is everything, or is it? Explorations on the meaning of psychological well-being. Journal of Personality and Social Psychology. 57(6), 1069–1081. 10.1037/0022-3514.57.6.1069.

[2] Ryan RM, Deci EL. (2001). On Happiness and Human Potentials: A Review of Research on Hedonic and Eudaimonic Well-Being. Annual Review of Psychology. 52(1), 141–166. doi: 10.1146/annurev.psych.52.1.141 11148302

[3] Brooks SK., Webster RK, Smith LE, Woodland L, Wessely S, Greenberg N, et al. (2020). The psychological impact of quarantine and how to reduce it: Rapid review of the evidence. Lancet. 395(10227), 912–920. doi: 10.1016/S0140-6736(20)30460-8 32112714 PMC7158942

[4] Pierce M., Hope H., Ford T., Hatch S., Hotopf M., John A., et al. (2020). Mental health before and during the COVID-19 pandemic: A longitudinal probability sample survey of the UK population. The Lancet. Psychiatry. 7(10), 883–892. doi: 10.1016/S2215-0366(20)30308-4 32707037 PMC7373389

[5] Xiong J., Lipsitz O., Nasri F., Lui L. M. W., Gill H., Phan L., et al. (2020). Impact of COVID-19 pandemic on mental health in the general population: A systematic review. Journal of Affective Disorders. 277, 55–64. doi: 10.1016/j.jad.2020.08.001 32799105 PMC7413844

[6] Vindegaard N., & Benros M. E. (2020). COVID-19 pandemic and mental health consequences: Systematic review of the current evidence. Brain, Behavior, and Immunity. 89, 531–542. doi: 10.1016/j.bbi.2020.05.048 32485289 PMC7260522

[7] Chevance A., Gourion D., Hoertel N., Llorca P.-M., Thomas P., Bocher R., et al. (2020). Ensuring mental health care during the SARS-CoV-2 epidemic in France: A narrative review. L’Encephale. 46(3), 193–201. doi: 10.1016/j.encep.2020.04.005 32370982 PMC7174154

[8] González-Blanco L., Dal Santo F., García-Álvarez L., de la Fuente-Tomás L., Moya Lacasa C., Paniagua G., et al. (2020). COVID-19 lockdown in people with severe mental disorders in Spain: Do they have a specific psychological reaction compared with other mental disorders and healthy controls? Schizophrenia Research, 223, 192–198. doi: 10.1016/j.schres.2020.07.018 32771308 PMC7381938

[9] Mengin A., Allé M. C., Rolling J., Ligier F., Schroder C., Lalanne L., et al. (2020). [Psychopathological consequences of confinement]. L’Encephale. doi: 10.1016/j.encep.2020.04.007 32370983 PMC7174176

[10] De Girolamo G., Dagani J., Purcell R., Cocchi A., & McGorry P. D. (2012). Age of onset of mental disorders and use of mental health services: Needs, opportunities and obstacles. Epidemiology and Psychiatric Sciences. doi: 10.1017/s2045796011000746 22670412

[11] Holmes E. A., O’Connor R. C., Perry V. H., Tracey I., Wessely S., Arseneault L., et al. (2020). Multidisciplinary research priorities for the COVID-19 pandemic: A call for action for mental health science. The Lancet. Psychiatry. 7(6), 547–560. doi: 10.1016/S2215-0366(20)30168-1 32304649 PMC7159850

[12] Yao H., Chen J.-H., & Xu Y.-H. (2020). Patients with mental health disorders in the COVID-19 epidemic. The Lancet. Psychiatry. 7(4), e21. doi: 10.1016/S2215-0366(20)30090-0 32199510 PMC7269717

[13] Seligman MEP (2002). Authentic Happiness: Using the New Positive Psychology to Realise Your Potential For Lasting Fulfilment. New York: Free Press.

[14] Haesebaert F., Haesebaert J., Zante E., Franck N. (2020). Who maintains good mental health in a locked-down country? A French nationwide online survey of 11,391 participants. Health & Place, 66, 102440. 10.1016/j.healthplace.2020.102440. PMC7490637 32947185

[15] Eysenbach G. (2004). Improving the Quality of Web Surveys: The Checklist for Reporting Results of Internet E-Surveys (CHERRIES). Journal of Medical Internet Research. 6(3). doi: 10.2196/jmir.6.3.e34 15471760 PMC1550605

[16] Tennant R., Hiller L., Fishwick R., Platt S., Joseph S., Weich S., et al. (2007). The Warwick-Edinburgh Mental Well-being Scale (WEMWBS): Development and UK validation. Health and Quality of Life Outcomes. 5, 63. doi: 10.1186/1477-7525-5-63 18042300 PMC2222612

[17] Trousselard M., Steiler D., Dutheil F., Claverie D., Canini F., Fenouillet F., et al. (2016). Validation of the Warwick-Edinburgh Mental Well-Being Scale (WEMWBS) in French psychiatric and general populations. Psychiatry Research. 245, 282–290. doi: 10.1016/j.psychres.2016.08.050 27565700

[18] Xiang Y.-T., Yang Y., Li W., Zhang L., Zhang Q., Cheung T., et al. (2020). Timely mental health care for the 2019 novel coronavirus outbreak is urgently needed. The Lancet. Psychiatry. 7(3), 228–229. doi: 10.1016/S2215-0366(20)30046-8 32032543 PMC7128153

[19] Fusar-Poli P., Salazar de Pablo G., De Micheli A., Nieman D. H., Correll C. U., Kessing L. V., et al. (2020). What is good mental health? A scoping review. European Neuropsychopharmacology. 31, 33–46. doi: 10.1016/j.euroneuro.2019.12.105 31901337

[20] García-Álvarez L., de la Fuente-Tomás L., García-Portilla M. P., Sáiz P. A., Lacasa C. M., Dal Santo F., et al. (2020). Early psychological impact of the 2019 coronavirus disease (COVID-19) pandemic and lockdown in a large Spanish sample. Journal of Global Health. 10(2). 10.7189/jogh.10.020505. PMC7567432 33110588

[21] González-Sanguino C., Ausín B., Castellanos M. A., Saiz J., López-Gómez A., Ugidos C., et al. (2020). Mental health consequences during the initial stage of the 2020 Coronavirus pandemic (COVID-19) in Spain. Brain, Behavior, and Immunity. 87, 172–176. doi: 10.1016/j.bbi.2020.05.040 32405150 PMC7219372

[22] Steardo L., & Verkhratsky A. (2020). Psychiatric face of COVID-19. Translational Psychiatry. 10. doi: 10.1038/s41398-020-00949-5 32732883 PMC7391235

[23] Millan M. J., Andrieux A., Bartzokis G., Cadenhead K., Dazzan P., Fusar-Poli P., et al. (2016). Altering the course of schizophrenia: Progress and perspectives. Nature Reviews. Drug Discovery. 15(7), 485–515. doi: 10.1038/nrd.2016.28 26939910

[24] Walker W. H., Walton J. C., DeVries A. C., & Nelson R. J. (2020). Circadian rhythm disruption and mental health. Translational Psychiatry. 10. doi: 10.1038/s41398-020-0694-0 32066704 PMC7026420

[25] Eime R. M., Young J. A., Harvey J. T., Charity M. J., & Payne W. R. (2013). A systematic review of the psychological and social benefits of participation in sport for children and adolescents: Informing development of a conceptual model of health through sport. The International Journal of Behavioral Nutrition and Physical Activity. 10, 98. doi: 10.1186/1479-5868-10-98 23945179 PMC3751802

[26] Pascoe M. C., Bailey A. P., Craike M., Carter T., Patten R., Stepto N. K., et al. (2020). Exercise interventions for mental disorders in young people: A scoping review. BMJ Open Sport & Exercise Medicine 6(1), e000678. doi: 10.1136/bmjsem-2019-000678 32426161 PMC7228557

[27] Zhang S. X., Wang Y., Rauch A., & Wei F. (2020). Unprecedented disruption of lives and work: Health, distress and life satisfaction of working adults in China one month into the COVID-19 outbreak. Psychiatry Research. 288, 112958. doi: 10.1016/j.psychres.2020.112958 32283450 PMC7146665

[28] Larson H. J. (2018). The biggest pandemic risk? Viral misinformation. Nature. 562, 309–309. doi: 10.1038/d41586-018-07034-4 30327527

[29] Shimizu K. (2020). 2019-nCoV, fake news, and racism. Lancet. 395(10225), 685–686. 10.1016/S0140-6736(20)30357-3. PMC7133552 32059801

[30] Zarocostas J. (2020). How to fight an infodemic. Lancet (London, England), 395(10225), 676. doi: 10.1016/S0140-6736(20)30461-X 32113495 PMC7133615

[31] Caleo G., Duncombe J., Jephcott F., Lokuge K., Mills C., Looijen E., et al. (2018). The factors affecting household transmission dynamics and community compliance with Ebola control measures: A mixed-methods study in a rural village in Sierra Leone. BMC Public Health, 18(1), 248. doi: 10.1186/s12889-018-5158-6 29439682 PMC5812186

[32] World Health Organization. (2020). Mental health and psychosocial considerations during the COVID-19 outbreak. World Health Organization. Retrieved from https://www.who.int/publications-detail-redirect/WHO-2019-nCoV-MentalHealth-2020.1.

[33] Rolland B., Haesebaert F., Zante E., Benyamina A., Haesebaert J., Franck N. (2020). Global Changes and Factors of Increase in Caloric/Salty Food Intake, Screen Use, and Substance Use During the Early COVID-19 Containment Phase in the General Population in France: Survey Study. JMIR Public Health and Surveillance, 6(3). 10.2196/19630. PMC7505683 32589149

[34] Bilbul M., Paparone P., Kim A. N., Mutalik S., Ernst C. L. (2020). Psychopharmacology of COVID-19. Psychosomatics, 61(5), 411–427. doi: 10.1016/j.psym.2020.05.006 32425246 PMC7232075

[35] Javelot H., Llorca P., Drapier D., Fakra E., Hingray C., Meyer G., et al. (2020). [Informations on psychotropics and their adaptations for patients suffering from mental disorders in France during the SARS-CoV-2 epidemic]. L’Encephale. doi: 10.1016/j.encep.2020.04.006 32376004 PMC7196532

[36] Vinkers C. H., van Amelsvoort T., Bisson J. I., Branchi I., Cryan J. F., Domschke K., et al. (2020). Stress resilience during the coronavirus pandemic. European Neuropsychopharmacology, 35, 12–16. doi: 10.1016/j.euroneuro.2020.05.003 32446705 PMC7211573

[37] Dillinger R. L., & Kersun J. M. (2020). Caring for caregivers: Understanding and meeting their needs in coping with first episode psychosis. Early Intervention in Psychiatry, 14(5), 528–534. doi: 10.1111/eip.12870 31452318

[38] Gupta M., & Bowie C. R. (2018). Family cohesion and flexibility in early episode psychosis. Early Intervention in Psychiatry, 12(5), 886–892. doi: 10.1111/eip.12384 27601077

[39] World Health Organization, Victorian Health Promotion Fundation, & University of Melbourne. (2005). Promoting mental health: Concepts, emerging evidence and practice (Summary Report) Geneva: World Health Organization. Retrieved from https://www.who.int/mental_health/publications/promoting_mh_2005/en/.

[40] UNAFAM. (2020). Baromètre UNAFAM (p. 23). Retrieved from https://www.unafam.org/actualites/lunafam-publie-son-premier-barometre-et-libere-les-maux-de-45-millions-de-proches.

[41] Ozbay F., Johnson D. C., Dimoulas E., Morgan C. A., Charney D., & Southwick S. (2007). Social support and resilience to stress: from neurobiology to clinical practice. Psychiatry (Edgmont (Pa.: Township)), 4(5), 35–40. 20806028 PMC2921311

